# Viral genetic clustering and transmission dynamics of the 2022 mpox outbreak in Portugal

**DOI:** 10.1038/s41591-023-02542-x

**Published:** 2023-09-11

**Authors:** Vítor Borges, Mariana Perez Duque, João Vieira Martins, Paula Vasconcelos, Rita Ferreira, Daniel Sobral, Ana Pelerito, Isabel Lopes de Carvalho, Maria Sofia Núncio, Maria José Borrego, Cornelius Roemer, Richard A. Neher, Megan O’Driscoll, Raquel Rocha, Sílvia Lopo, Raquel Neves, Paula Palminha, Luís Coelho, Alexandra Nunes, Joana Isidro, Miguel Pinto, João Dourado Santos, Verónica Mixão, Daniela Santos, Silvia Duarte, Luís Vieira, Fátima Martins, Jorge Machado, Vítor Cabral Veríssimo, Berta Grau, André Peralta-Santos, José Neves, Margarida Caldeira, Mafalda Pestana, Cândida Fernandes, João Caria, Raquel Pinto, Diana Póvoas, Fernando Maltez, Ana Isabel Sá, Mafalda Brito Salvador, Eugénio Teófilo, Miguel Rocha, Virginia Moneti, Luis Miguel Duque, Francisco Ferreira e Silva, Teresa Baptista, Joana Vasconcelos, Sara Casanova, Kamal Mansinho, João Vaz Alves, João Alves, António Silva, Miguel Alpalhão, Cláudia Brazão, Diogo Sousa, Paulo Filipe, Patrícia Pacheco, Francesca Peruzzu, Rita Patrocínio de Jesus, Luís Ferreira, Josefina Mendez, Sofia Jordão, Frederico Duarte, Maria João Gonçalves, Eduarda Pena, Claúdio Nunes Silva, André Rodrigues Guimarães, Margarida Tavares, Graça Freitas, Rita Cordeiro, João Paulo Gomes

**Affiliations:** 1 Genomics and Bioinformatics Unit, Department of Infectious Diseases, National Institute of Health Doutor Ricardo Jorge (INSA), Lisbon, Portugal; 2 Epidemiology and Statistics Division, Directorate-General of Health, Lisbon, Portugal; 3 Pathogen Dynamics Group, Department of Genetics, University of Cambridge, Cambridge, United Kingdom; 4 Public Health Emergency Centre, Directorate-General of Health, Lisbon, Portugal; 5 Emergency Response and Biopreparedness Unit, Department of Infectious Diseases, National Institute of Health Doutor Ricardo Jorge (INSA), Lisbon, Portugal; 6 National Reference Laboratory for Sexually Transmitted Infections, Department of Infectious Diseases, National Institute of Health Doutor Ricardo Jorge (INSA), Lisbon, Portugal; 7 Biozentrum, University of Basel, Basel, Switzerland; 8 Swiss Institute of Bioinformatics, Basel, Switzerland; 9 Veterinary and Animal Research Centre (CECAV), Faculty of Veterinary Medicine, Lusófona University, Lisbon, Portugal; 10 Technology and Innovation Unit, Department of Human Genetics, National Institute of Health Doutor Ricardo Jorge (INSA), Lisbon, Portugal; 11 Technical Board, National Institute of Health Doutor Ricardo Jorge (INSA), Lisbon, Portugal; 12 Department Coordination, Department of Infectious Diseases, National Institute of Health Doutor Ricardo Jorge (INSA), Lisbon, Portugal; 13 Public Health Unit, ACES Cascais, ARSLVT, Cascais, Portugal; 14 ECDC Fellowship Programme, Field Epidemiology path (EPIET), European Centre for Disease Prevention and Control (ECDC), Solna, Sweden; 15 Directorate of Information and Analysis, Directorate-General of Health, Lisbon, Portugal; 16 Comprehensive Health Research Centre (CHRC), Escola Nacional de Saúde Pública, Universidade NOVA de Lisboa, Lisbon, Portugal; 17 Serviço de Dermatovenereologia, Consulta de DST, Centro Hospitalar Universitário de Lisboa Central, Lisbon, Portugal; 18 Serviço de Doenças Infeciosas, Hospital de Curry Cabral, Centro Hospitalar Universitário de Lisboa Central, Lisbon, Portugal; 19 Instituto Gulbenkian de Ciência, Oeiras, Portugal; 20 Unidade de Doenças Sexualmente Transmissíveis da Lapa, Lisbon, Portugal; 21 GAT - Grupo de Ativistas em Tratamentos, Av. Paris, Lisbon, Portugal; 22 GAT - Grupo de Ativistas em Tratamentos, Intendente, Lisbon, Portugal; 23 Serviço de Doenças Infeciosas e Medicina Tropical, Hospital de Egas Moniz, Centro Hospitalar de Lisboa Ocidental, Lisbon, Portugal; 24 Serviço de Dermatovenereologia, Hospital Garcia de Orta, Almada, Portugal; 25 Dermatology Department, Centro Hospitalar Universitário Lisboa Norte EPE, Lisbon, Portugal; 26 Dermatology Research Unit (PFilipe Lab), Instituto de Medicina Molecular João Lobo Antunes, University of Lisbon, Lisbon, Portugal; 27 Dermatology University Clinic, Faculty of Medicine, University of Lisbon, Lisbon, Portugal; 28 Serviço de Infeciologia, Hospital Professor Doutor Fernando Fonseca, Amadora, Portugal; 29 Serviço Infeciologia do CHUP, Largo Professor Abel Salazar, Porto, Portugal; 30 Serviço de Doenças Infeciosas, Hospital Pedro Hispano – ULS Matosinhos, Matosinhos, Portugal; 31 Serviço de Doenças Infeciosas, Centro Hospitalar Universitário de São João, Porto, Portugal; 32 National Health Authority, Directorate-General of Health, Lisbon, Portugal

**Keywords:** Viral genetics, Viral infection

## Abstract

Pathogen genome sequencing during epidemics enhances our ability to identify and understand suspected clusters and investigate their relationships. Here, we combine genomic and epidemiological data of the 2022 mpox outbreak to better understand early viral spread, diversification and transmission dynamics. By sequencing 52% of the confirmed cases in Portugal, we identified the mpox virus sublineages with the highest impact on case numbers and fitted them into a global context, finding evidence that several international sublineages probably emerged or spread early in Portugal. We estimated a 62% infection reporting rate and that 1.3% of the population of men who have sex with men in Portugal were infected. We infer the critical role played by sexual networks and superspreader gatherings, such as sauna attendance, in the dissemination of mpox virus. Overall, our findings highlight genomic epidemiology as a tool for the real-time monitoring and control of mpox epidemics, and can guide future vaccine policy in a highly susceptible population.

## Abstract (web summary)

An analysis of viral genomes and epidemiological data from the 2022 mpox outbreak in Portugal sheds light on the heterogeneity of early transmission events, with national and international linkages.

## Main

Mpox is a viral zoonosis caused by mpox virus (MPXV), a member of the genus Orthopoxvirus that also includes variola virus (which causes smallpox), vaccinia virus, camelpox virus and cowpox virus, all of which are pathogenic to humans^1–5^. MPXV was discovered in 1958, when a non-lethal rash disease broke out in captive cynomolgus monkeys in Copenhagen, Denmark^6^. The first case in humans was reported in 1970 in the Democratic Republic of Congo, where a 9-month-old infant presented with what appeared to be an early-stage smallpox rash^7^. MPXV infection is often caused by spill-over events from animals (such as small rodents and non-human primates) to humans^1–5^. However, mpox can also be transmitted from person to person through direct contact with lesions, body fluids and respiratory secretions, or contact with contaminated material^1,4,8^.

Mpox is endemic in West and Central Africa, where several outbreaks have occurred in recent decades^1–4,9–12^. Nevertheless, until 2022, only small clusters or sporadic cases of mpox had been recorded outside of endemic regions. These cases were generally travel-related and linked to countries with endemic mpox^1–4,12–17^ or related to imported small mammals^18^, but had limited subsequent human-to-human sustained transmission. Presently, a large multi-country mpox outbreak is ongoing worldwide. The first cases were reported in May 2022, with 88,600 laboratory-confirmed cases and 152 deaths being reported in 113 member states across all six World Health Organization (WHO) regions by 2 August 2023 (ref. ^19^). On 23 July 2022, the WHO Director-General declared this outbreak a public health emergency of international concern^20^. The outbreak has disproportionately affected men who have sex with men (MSM), and MSM with mpox frequently had skin lesions in the anogenital area, suggesting the amplification of transmission through sexual networks^21–23^.

MPXV genomes from the 2022 outbreak belong to clade IIb^24,25^ according to the recently proposed nomenclature^25^. This clade, within the formerly designated ‘West African’ clade, is characterized by less severe symptoms and a lower lethality rate than clade I (formerly ‘Congo Basin’ clade)^25,26^. Additionally, as clade IIb viruses have shown sustained human-to-human transmission, a new subclade was designated ‘hMPXV1’ (ref. ^25,27^). Within this subclade, a hierarchy of lineages (starting with ‘A’) similar to SARS-CoV-2 lineage labels was proposed. The MPXV causing the current outbreak was classified as lineage B.1, which has been further subdivided into sublineages B.1.1, B.1.2, and so on (https://github.com/mpxv-lineages)^25^. The first comparative genomic analyses of these genomes revealed that the MPXV causing the 2022 multi-country outbreak shares a recent common ancestor with MPXV sequences connected to Nigeria (A.1 lineage)^24^, where a large outbreak occurred in 2017 and 2018 (ref. ^12,16,28^). More recently, other hMPXV1 lineages (for example, A.2.1, A.2.2, A.2.3 or A.3) have been sporadically detected in countries without endemic mpox from a wide latitude (for example, the United States, Vietnam, Egypt and the United Kingdom), supporting the aforementioned link to the epidemic in Nigeria and that other human-to-human transmission chains are probably ongoing besides the recognized large B.1 outbreak^27,29^. However, the number of sequences reported throughout the years (until 2022) is still very limited, hindering the establishment of precise origins, evolutionary routes and clone dissemination. The sequences collected during the 2022 outbreak diverge by around 50 single-nucleotide polymorphisms (SNPs) from pre-outbreak sequences^24^. This indicates an unexpectedly high substitution rate for the slow-evolving double-stranded DNA Orthopoxvirus. In-depth mutational analysis suggested the action of host APOBEC3 (apolipoprotein B mRNA-editing catalytic polypeptide-like 3) enzymes in viral evolution (owing to the mutating bias of these SNPs: GA>AA and TC>TT base substitutions) and enabled us to estimate that the introduction of hMPXV1 into the human population occurred around 2016, probably followed by ‘silent’ human-to-human transmission^27^. Signs of potential MPXV–human adaptation in the microevolution of the current outbreak were also investigated early on^24,30,31^, but the adaptive value of the APOBEC3 hypermutation in the long term is still uncertain^27^.

Since the beginning of the 2022 outbreak, and mirroring the open science practices observed in the case of SARS-CoV-2, researchers from several countries immediately started to share MPXV genomes in public databases (for example, GISAID, GenBank), and existing platforms (such as Nextstrain (https://nextstrain.org/monkeypox/hmpxv1) and GenSpectrum (https://mpox.genspectrum.org/)) were rapidly adapted. This collaborative environment facilitated the execution of phylogenetics-based spatiotemporal studies and the surveillance of the genetic variability of MPXV by tracking its spread and assessing evolutionary traits potentially linked to its adaptation to the human host or immune evasion^24,27,30–32^. Since the first confirmed cases in Portugal on 17 May 2022 (ref. ^24,33^), the country has embarked on a major effort of to sequence the MPXV and share data, and has been able to obtain the genome sequences for more than 50% of all reported mpox cases in the country, as of January 2023. In addition, in Portugal, MPXV infection was included in the National Epidemiological Surveillance System (SINAVE), which gathers demographic, clinical, epidemiological and laboratory data of all mandatory notifiable diseases. Nevertheless, even with comprehensive epidemiological surveillance systems and wide availability of diagnostics tests, how transmission occurs remains uncertain. Mathematical models are particularly useful to understand partly observed infectious disease phenomena^34^. In particular, epidemic models like the SEIR (Susceptible-Exposed-Infectious-Recovered) model divide the population into different groups based on infection status and can be used to estimate time-dependent parameters such as the time-varying reproduction number (*R*_*t*_) and the expected number of susceptible, infected and recovered cases throughout the time series^35^. To date, the early transmission dynamics of the international MPXV outbreak have not been well characterized. Here, we combined epidemiological and genomic data to better understand the transmission dynamics of MPXV, providing evidence that can help to tailor public health messages and guide vaccine policy.

## Results

### Cases and sequence sampling

**Fig. 1:**
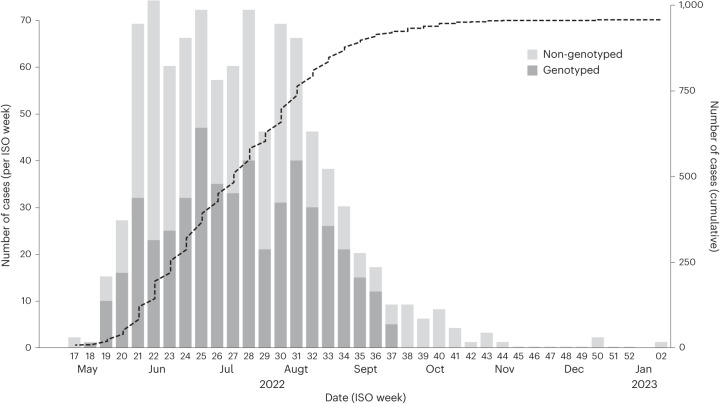
Cumulative and weekly cases of mpox, and viral genome sequence sampling in Portugal, as of 10 January 2023. The bars depict the numbers of cases distributed by date of collection (ISO week) of MPXV-positive samples and are colored according to the availability of viral genotyping data. The dashed line shows the cumulative number of cases.

The first mpox cases in Portugal were laboratory-confirmed on 17 May 2022, and the epidemic peaked around 17 July 2022, followed by a decreasing trend in the number of cases. A total of 951 cases were confirmed in Portugal, as of 10 January 2023 (Fig. 1). To understand the genomic epidemiology of MPXV in Portugal during the 2022 multi-country outbreak, viral genome sequences were obtained from 495 individuals with a PCR-positive test. The dates of sample collection of the 495 genotyped cases spanned from 4 May (ISO week 18) to 16 September (ISO week 37) 2022, representing a large sequence sampling of 54.2% (495 out of 914) of all confirmed mpox cases in Portugal during this period (corresponding to 52.1% of total confirmed cases, as of 10 January 2023) (Fig. 1). Demographic variables of sex and age were available for 447 out of the 495 studied mpox cases. Most of these individuals (99.1%; 443 out of 447) identified as male. A majority were in the age bracket 30–39 years (44.1%; 197 out of 447), followed by the age brackets 20–29 years (28.4%; 127 out of 447) and 40–49 years (20.4%; 91 out of 447). Human immunodeficiency virus (HIV) infection status was reported as positive in 42.9% of individuals with mpox who provided their status (168 out of 392). Among males who reported their sexual orientation (*n* = 344), 96.5% (332 out of 344) self-identified as MSM. To better understand the introduction and transmission dynamics of MPXV in Portugal, epidemiological data were collected with particular focus on travel history, exposure settings and transmission routes that could be complemented by the available genotyping data (Supplementary Table 1).

### Transmission dynamics

**Fig. 2:**
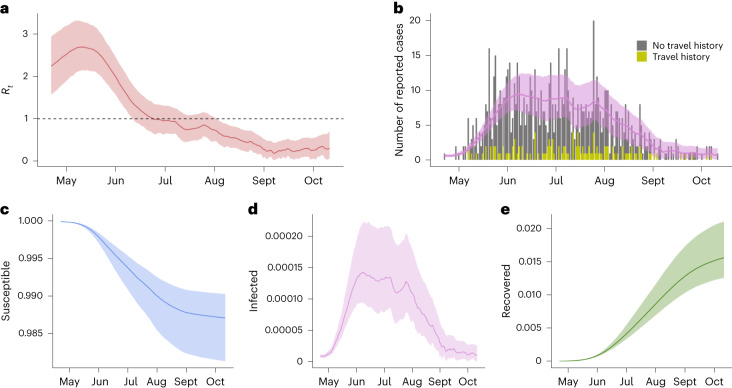
Model parameter estimates and model fit of the MPXV outbreak in Portugal, April–October 2022. **a**, The estimated *R*_*t*_ using Hamiltonian Monte Carlo No-U-Turn sampling. The solid red line shows the median *R*_*t*_ estimates for each time point. The red band shows the 95% CrI of *R*_*t*_. The horizontal black dashed line at value 1 shows the threshold for continued transmission. **b**, The model fit against the epidemic curve of MPXV cases by travel history data (reported during the incubation period). The solid pink line shows the estimated median of case numbers for each time point and the pink band the corresponding 95% CrI. **c**–**e**, The proportion of the MSM population estimated to be susceptible (**c**), infected (**d**) and recovered (**e**) from MPXV throughout the time series using an incubation period of 5.6 days and with ten infection seeds. The corresponding colored bands show the 95% CrI. MPXV case notification data (*n* = 865) were obtained from SINAVE. Sensitivity analyses across different priors and assumptions are shown in Supplementary Figs. 1, 2 and 3.

To understand the transmission intensity and estimate key epidemiological parameters of this outbreak in Portugal, we used case incidence data to fit a time-discrete SEIR model (Fig. 2). We observed an estimated *R*_*t*_ of 2.25 (95% credible interval (CrI), 1.59–2.95) at the beginning of the time series (earliest reported onset of symptoms, 22 April 2022), increasing to a maximum of 2.70 (95% CrI, 2.11–3.30) on 10 May 2022 (Fig. 2a). We estimated a subsequent decline in *R*_*t*_, falling below 1 at the end of June 2022. The model accounted for travel history and was able to reconstruct well the true number of cases (Fig. 2b), with an estimated reporting rate of 0.62 (95% CrI, 0.43–0.83). We also estimated the proportions of the MSM population in Portugal that were susceptible (Fig. 2c), infected (Fig. 2d) and recovered (Fig. 2e). The model calculated that 1.3% (*n* = 1,370; 95% CrI, 818–2,228) of MSM were infected during this period, with 98.7% (95% CrI, 98.1–99.0) of the MSM population still susceptible to infection at the end of this time series. The model estimated the epidemic peak, defined as the highest daily incidence of confirmed cases, to occur on 8 June 2022 with 15 infections (95% CrI, 10–24), before faltering slightly and subsequently declining rapidly from the end of July 2022. In parallel, we conducted a sensitivity analysis with no travel history, across different infectious periods of 14, 21 and 28 days ($\sigma$) ∈ {0.071, 0.048, 0.036} and with different infection seeds and found largely consistent model estimates (Supplementary Figs. 1, 2 and 3).

### Identification of subclusters

Of the 495 MPXV genome sequences from Portugal analyzed in the present study, 494 belonged to the B.1 lineage (and sublineages) associated with the large multi-country outbreak. One sequence (PT0428; date of collection, 1 August 2022) belonged to sublineage A.2.3 (not linked to the large outbreak), thus representing an independent introduction of the virus into the country. The individual to whom these data relate self-identifies as MSM, and reported travel history during the incubation period. Although the individual is from a country endemic for mpox in the West Africa region, local health authorities were unable to determine the specific countries visited by the individual during the relevant period. As of 2 November 2022, the publicly available Nextstrain hMPXV1 phylogenetic tree (https://nextstrain.org/groups/neherlab/PT-MPXV-transmission/2022-11-02) included 2,714 outbreak sequences (18.2% from Portugal) from 28 countries across Europe, Asia, Africa, Oceania, South America and North America (Supplementary Table 1). Of the 2,714 outbreak sequences, 377 were phylogenetically placed at the outbreak basal level (that is, identical consensus sequences with no extra mutations). This considerably high number of ‘root’ sequences was not unexpected because viral dissemination at the global level might have occurred very rapidly, probably triggered by multiple superspreader events, and led to a rapid clonal expansion with less opportunity for mutation diversification. Most of the sequences, 2,126 (78.3%), belonged to genetic subclusters, defined as subbranches with at least two sequences diverging from the 2022 outbreak basal level by at least one SNP. This subcluster definition (that is, one SNP above the outbreak ‘root’) is aligned with criteria being applied to designate international MPXV sublineages according to the nomenclature proposed in https://github.com/mpxv-lineages. A total of 182 genetic subclusters were identified in the global tree, with 52 of them (78.3%) including at least two Portuguese sequences (hereafter called ‘Portuguese subclusters’) (Supplementary Table 2). The Portuguese subclusters comprised 66.6% (329 out of 494) of sequences from Portugal, meaning that around two-thirds of the mpox cases could be, at least phylogenetically, linked to another case(s) detected in Portugal. In total, 96 out of the 494 Portuguese sequences (19.4%) were phylogenetically placed at the outbreak basal level. The remaining 69 sequences (14.0%) formed ‘singleton’ branches (*n* = 49) or were integrated within international clusters with a single Portuguese sequence (*n* = 20) (Supplementary Tables 1 and 2).

### Size, timespan and international ‘linkage’ of Portuguese subclusters

**Fig. 3:**
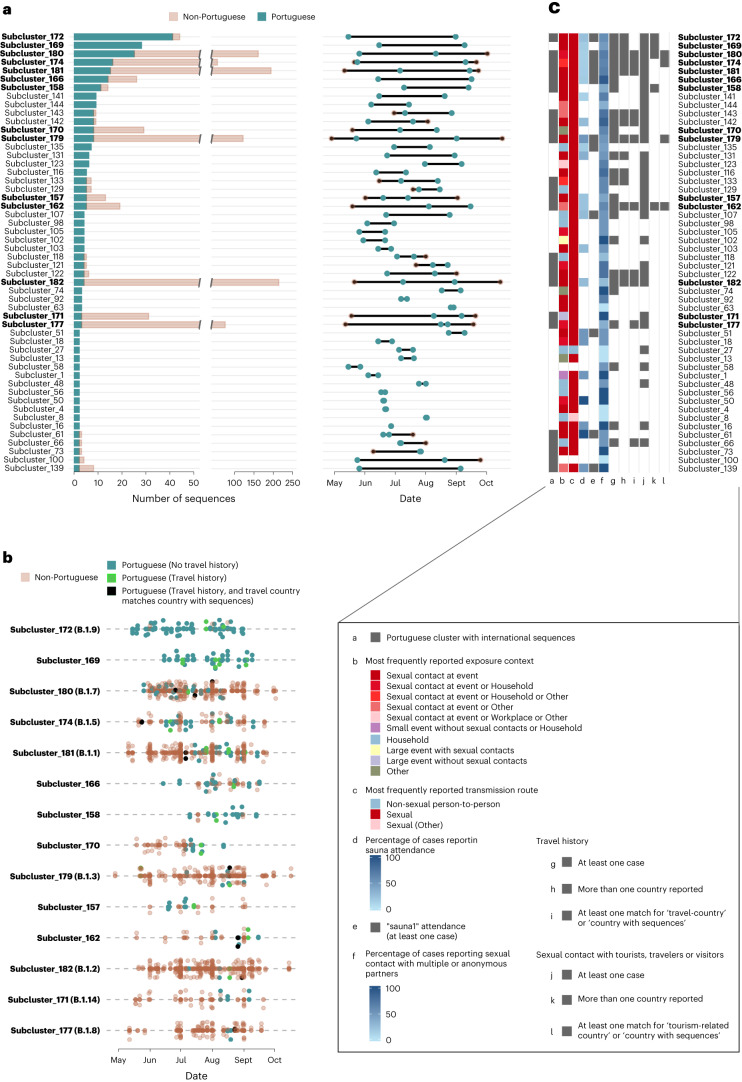
Spatiotemporal landscape, international linkage and transmission dynamics of the main viral sublineages of MPXV detected in Portugal during the 2022 multi-country mpox outbreak. **a**, Characterization of Portuguese genetic subclusters (that is, subclusters including at least two Portuguese sequences) in terms of the number of sequences (and inclusion of sequences from other countries), and their timespan at national and international levels. **b**, Detail of the temporal landscape of the sequences of main MPXV sublineages (that is, more than ten sequences globally; bold in **a**). Portuguese cases (in green) reporting travel history are highlighted in black or bright green when the travel-related country matches or does not match a country with sequences in the same cluster, respectively. **c**, Characterization of the Portuguese genetic subclusters according to the most frequently reported transmission routes and exposure context (excluding cases with no data available), and to potential international linkage based on travel history and/or sexual contact with tourists, travelers or visitors in the 21 days before symptom onset.

The Portuguese subclusters showed considerable diversity in terms of size, timespan and inclusion of sequences from other countries (Fig. 3a). Seven Portuguese subclusters contained at least ten sequences from Portugal, with cluster 172 (corresponding to the internationally designated sublineage B.1.9) including the highest number of Portuguese sequences (41 Portuguese and 3 international sequences). In particular, 25 out of the 52 Portuguese subclusters (48.1%) included international sequences (Fig. 3a and Supplementary Table 2). The observation that 27 Portuguese subclusters (51.9%) exclusively included sequences from Portugal should be interpreted with caution because of the high discrepancies in sequence sampling between countries. It also suggests that, as expected, some ‘sublineages’ (that is, genetic subclusters) had a more restricted circulation in Portugal, while others showed considerable international spread. Among the latter, we highlight the considerable circulation in Portugal of the internationally designated sublineages B.1.1 (*n* = 15), B.1.5 (*n* = 16) and B.1.7 (*n* = 25), which are among the five largest Portuguese subclusters (Fig. 3a and Supplementary Table 2). Nonetheless, in contrast to sublineage B.1.9, with 93% of sequences from Portugal, all of these international lineages included less than 30% Portuguese sequences, as of 2 November 2022 (Fig. 3a and Supplementary Tables 1 and 2). Other internationally designated sublineages detected in Portugal were B.1.2, B.1.3, B.1.8, B.1.11 and B.1.14, and these included even less Portuguese sequences (each with less than ten Portuguese sequences and/or with Portuguese sequences representing less than 10% of the cluster size). For instance, the largest cluster at the global level, corresponding to sublineage B.1.2, included 214 sequences, with only four sequences (1.9%) being from Portugal (Fig. 3a and Supplementary Tables 1 and 2).

In addition, we assessed the timespan of the subclusters, that is, the time (in days) between the earliest and latest detection of a given sublineage at national and international levels (Fig. 3a,b and Supplementary Table 2). At the international level, as of 2 November 2022, the largest time interval (172 days) was observed for sublineage B.1.3, which has been detected in 13 countries since 28 April 2022 (Fig. 3b). It is noteworthy that the earliest occurrence of several Portuguese subclusters that involved international sequences was observed in a Portuguese case, suggesting that the emergence of some outbreak sublineages (or, at least, the early dissemination of some sublineages at a global level) occurred in Portugal (Fig. 3a). At the national level, 24 out of the 52 Portuguese subclusters included mpox cases detected in a time interval equal to or above 30 days (Fig. 3a and Supplementary Table 2). The largest time interval between two Portuguese cases of the same cluster was observed for cluster 174 (corresponding to the international sublineage B.1.5), with a total of 109 days (between ISO weeks 21 and 36, 2022) (Fig. 3b).

Given the estimated substitution rate of the outbreak-causing MPXV^27^ and its spread through sexual networks^1,22^, it is worth noting that a genetic subcluster does not necessarily represent a sustained transmission chain. To address this challenge of detecting multiple introductions, we assessed whether individuals within the same subcluster reported travel history (within the incubation period of MPXV) to different countries and then assessed whether those countries were represented by sequences in the same subcluster. Travel history was reported for 85 out of 494 cases (17.2%), with 43 of them belonging to subclusters with international sequences (Supplementary Tables 1 and 2). The names of travel-related countries (that is, countries from the travel history inquiry, known for 84 out of 85 cases) were reported by at least one case for 25 Portuguese subclusters (17 having international sequences) and 6 international subclusters with a single Portuguese sequence. Not unexpectedly, for 14 Portuguese subclusters, more than one country of travel was reported by different individuals whose sequences were in the same subcluster, thus providing strong evidence for more than one introduction of those 14 sublineages into the country (Fig. 3c and Supplementary Table 2). For instance, this scenario was identified for the four internationally designated sublineages showing the highest circulation in Portugal (B.1.1, B.1.5, B.1.7 and B.1.9). Most notably, for 11 subclusters, we found at least one match between a travel-related country and a country with sequences in those subclusters, with that country being the most or second most represented in terms of the number of sequences for seven subclusters (Fig. 3c and Supplementary Table 2).

In addition to the inclusion of travel history, epidemiological data on sexual contact with tourists, travelers or visitors was reported in 101 out of 494 cases (20%) (Supplementary Table 1) and could indirectly inform about potential introductions or reintroductions of the virus into Portugal. Most of these cases, 50 out of 101 (49.5%), belonged to subclusters with international sequences, namely 20 Portuguese subclusters and 6 international subclusters with a single Portuguese sequence. The name of the tourism-related country (that is, countries linked to sexual contacts with tourists, travelers or visitors) was specified by 50 individuals. In total, sexual contact with tourists from a known country was confirmed by at least one Portuguese individual for 21 subclusters (13 with international sequences). Similarly to the scenario observed after the inclusion of travel history data, we found subclusters with more than one tourism-related country (reported by different individuals) in the same cluster (potentially reflecting additional independent introductions), as well as subclusters in which a given tourism-related country matched a country with sequences in that cluster (Fig. 3c and Supplementary Table 2).

### Transmission routes and exposure context of Portuguese subclusters

Detailed epidemiological data on the most probable transmission routes and on possible exposure in the 21 days before symptom onset were available for most of the studied cases (399 and 318 out of the 494 cases, respectively) (Supplementary Table 1). Sexual contact was reported as a possible route of transmission in 95.2% (380 out of 399) of cases, followed by non-sexual person-to-person transmission (3.3%; 13 out of 399), healthcare-associated transmission (0.5%; 2 out of 399) and ‘other’ (1.0%; 4 out of 399). Regarding the exposure context, most cases reported exposure at events involving sexual contacts, either small (such as private party or club, or sauna) (49.7%; 158 out of 318) or large (such as festivals) (2.8%; 9 out of 138). Household exposure was reported by 84 out of 318 cases (26.4%), 79 of which reported sexual transmission. Additional exposure contexts included small (3.5%) and large (1.6%) events without sexual contacts, workplace (0.9%), healthcare services (0.6%) and ‘other’ (14.1%) (Supplementary Table 1).

Within each Portuguese subcluster, small events involving sexual contacts were reported as the most frequent exposure context in 34 subclusters, followed by household exposure (most frequently reported in ten small subclusters with two to seven cases per subcluster) (Fig. 3c and Supplementary Table 2). The in-depth epidemiological investigation also recorded sauna attendance in the 21 days before symptom onset for 76 out of the 494 studied individuals. Notably, 71 out of the 76 cases reporting ‘sauna’ (93%) were phylogenetically placed at the outbreak basal level (‘root’) (*n* = 22) or were part of subclusters (*n* = 49), with 33 belonging to subclusters with international sequences (Supplementary Table 1). In total, 23 out of the 52 Portuguese subclusters included at least one case reporting ‘sauna’ (Fig. 3c and Supplementary Table 2). In particular, sauna attendance was reported in at least one case for the top six subclusters in terms of the number of Portuguese sequences. We could trace back the attendance of 28 of the 76 individuals who reported sauna attendance to one particular sauna (‘sauna1’) located in the Lisbon and Tagus Valley geographic region. These cases were found across 11 Portuguese subclusters, eight of which included international sequences (Fig. 3c). Sexual contacts with multiple or anonymous individuals were also reported by 283 out of 494 cases (57.3%), with nine subclusters exclusively involving cases that reported this variable (Fig. 3c and Supplementary Table 2).

## Discussion

The estimated substitution rate of the MPXV that caused the 2022 outbreak (around six substitutions per genome per year)^27^ and its transmission characteristics, associated with sexual networks and superspreader events providing the opportunity for rapid outbreak dissemination^21–23^, challenge the identification and tracking of singular transmission chains, as well as the distinction between continuous within-country transmission from multiple introductions. Still, our transmission model and genetic clustering analysis with extensive epidemiological data integration provide evidence regarding the transmission dynamics of MPXV.

Despite the complexity of identifying the epidemiological parameters of MPXV in this outbreak, we fitted an SEIR model accounting for travel history and obtained a reasonable fit. Owing to the long infectious period of MPXV^2^, we expected that *R*_*t*_ would fall below 1 before cases start declining, as observed in this outbreak. The fast decline of the *R*_*t*_ in Portugal can be explained by three main factors: contact tracing procedures, behavioral changes and vaccination. Contact tracing procedures were implemented as early as May 2022, when the first cases were reported. Intimate contacts and sexual partners of cases were identified, when possible, and were advised to stay in isolation for the maximum duration of the incubation period. During the pre-smallpox-vaccination period, in June 2022, the public health strategy focused on mobilizing the MSM population for prevention and control measures and on identifying groups with high risk of MPXV transmission, particularly those who would have a high number of sexual partners. This particular group of the MSM population might have played a key role in viral transmission in this outbreak^36,37^, as supported by the present study. Targeted public health messages to the MSM population and community stakeholders working in the field of sexually transmitted diseases (STDs) probably led to behavior changes, as seen by the rapid decrease of the *R*_*t*_. Following the approval of the smallpox vaccine for emergency use in the mpox outbreak, ring vaccination started in Portugal in July 2022, consolidating the decrease in transmission risk. The difference between the estimated and the observed epidemic peaks probably translates to a lag of reporting. One of the unique features of this outbreak is that most cases self-identify as MSM. We estimate that only 1.3% of the MSM population in Portugal^38^ was infected with the virus, leaving an almost entirely susceptible MSM population with the potential for future epidemics. This could be corroborated by a targeted seroprevalence study in Portugal, as well as in other high incidence-reporting countries. Recent studies have suggested the role of both cryptic and asymptomatic transmission, which could explain, partly, the proportion of cases not observed and not reported^33,39–41^. This model emphasizes the value of transmission models, which enables us to estimate the true underlying size of the epidemic, accounting for reporting probabilities as well as susceptible population proportions.

Our transmission model has potential limitations in the assumptions adopted, which are important to consider when interpreting the results. Firstly, we assumed a homogeneous mixing of the entire MSM population living in Portugal, and we did not include network data among the MSM population because of the incompleteness of these data in the inquiries. Secondly, we relied on laboratory-confirmed case data as an indicator of epidemic size and used travel history as an approximation for the imported cases. Lastly, the model does not account for the possibility that some exposed individuals are perhaps less susceptible to the MPXV (for example, smallpox-vaccinated individuals), and the low number of vaccinated individuals during the period of this time series (less than 500) hampered the inclusion of the effect of vaccination on these dynamics.

Regarding the genomic epidemiological analysis, we were able to identify the B.1 sublineages (that is, genetic subclusters) with the highest impact on the mpox epidemic in Portugal and assess whether their circulation seemed to be higher in Portugal (owing to the lack of international sequences) or revealed multi-country dissemination (Fig. 3). Moreover, the inclusion of travel data, as well as data on sexual contacts with tourists, travelers or visitors, complemented the phylogenetic data to support the existence of multiple introductions of the same sublineage, even though different scenarios were observed. For example, sublineage B.1.9, which had intense circulation in Portugal since the beginning of the epidemic (Fig. 3), was found to have been reintroduced at a later stage after the epidemic peak. By contrast, other sublineages seemed to be introduced multiple times throughout the epidemic, although each introduction probably seeded limited transmission chains (for example, B.1.5 and B.1.7). Supporting the reliability of the disclosed international linkage, we found a link between travel-related or tourism-related countries and the countries with (a high proportion of) sequences in those subclusters. For instance, we found one travel-related introduction of sub-lineage B.1.2 from the United States, and this country represented around half (105 out of 214) of all B.1.2 sequences reported worldwide during the study period. In another example, Germany was the most frequently represented country (114 out of 161 sequences) within cluster 181 (corresponding to sublineage B.1.1), which was consistent with the travel history reported by one Portuguese case linked to that cluster. Still, it is noteworthy that travel history data also allowed the identification of other potential independent B.1.1 introductions from Brazil, Canada and Italy, which corroborates the complexity of such inferences, even with the vast epidemiological data and high sequence sampling rates (Supplementary Tables 1 and 2). Also, in a few instances, the lack of matches could have been underestimated because of the low number of sequences available in GenBank from some countries. For example, several cases across different subclusters reported travel history to Spain and Brazil, but only a few matches were found.

Attendance at small venues involving sexual contacts, namely saunas (in particular ‘sauna1’), was found to be among the most reported exposure contexts within the studied population, which is consistent with previous reports^1,21,22,32,42,43^. We cannot directly point to saunas as the main triggers for the extensive dissemination of MPXV, as it is not possible to ascertain whether these locations were the actual settings of transmission for all of these cases. However, our study clearly supports the important role of such potential superspreader events in outbreak dissemination^21,22,38^, as these cases were frequently integrated into large subclusters (for example, they were reported for the six largest subclusters) and were commonly found across subclusters with international sequences (for example, almost half of the cases reporting sauna attendance were linked to these subclusters). From another perspective, the potential epidemiological linkage of a particular setting (for example, ‘sauna1’) to several subclusters clearly shows the difficulties of untying the complex transmission networks associated with this multi-country outbreak. These challenges are highlighted not only by the considerably high number of cases reporting sexual contact with multiple or anonymous individuals, but also because almost all subclusters (49 out of 52) involved cases attending different hospitals or STD clinics. For instance, each of the five largest subclusters involved cases from at least 11 different hospitals or STD clinics, with potential negative impact on epidemiological data collection, despite the existence of standardized case investigation forms. Furthermore, these challenges cannot be disconnected from the intrinsic subjectivity associated with the interviews, which are dependent on both recall bias and the participants’ willingness to provide the requested information, considering the exposure context (sexual networks). Future efforts to develop methods to improve the collection of this information while protecting individuals’ privacy are warranted to increase the added value of genomic epidemiology. This is well reflected by the fact that the epidemiological inquiries were able to collect information about person-to-person contact for only 13 pairs of individuals among the 494 studied cases. This information is pivotal to complement the genomic data to track transmission. Indeed, the phylogenetic data clearly discard a direct or indirect epidemiological connection between the cases only for two out of the 13 pairs (Supplementary Table 1). This concordance (that is, most known contacts were confirmed in the same genetic subclusters) suggests that our clustering analysis might provide a linkage between most cases within the same cluster. We are aware that because of the large size of the dataset and MPXV genome, occasional sequence or phylogenetic artifacts (for example, reversions, spurious mutations or false homoplasies) might insert inconsistencies into the clustering. Still, our conservative clustering approach and inspection of the Portuguese data suggests that this effect may be minimal, reinforcing the presented global clustering scenario, which would be difficult to uncover with only epidemiological data. An unexpected finding was the detection of a non-outbreak-related sequence (PT0428) from sublineage A.2.3. This case, also self-identified as MSM, could be linked to the West Africa region and parallels other recent detections of non-B.1 lineages across the world^27^. By unveiling concurrent human-to-human transmission and broad dissemination of multiple hMPXV-1 lineages (outside the B.1 outbreak), these recent findings corroborate the idea that the historical paradigm of MPXV ecology, evolution and epidemiology has changed, posing new challenges for the prevention and control of mpox^24,27,29,30^.

By taking advantage of a large sequence sampling and the collection of vast epidemiological data, we shed light on the introductions and transmission dynamics of MPXV in Portugal. As one of the countries that reported the first cases and, similarly to the United Kingdom^42^ and Spain^38^, one of the most affected countries during the early stages of the 2022 outbreak^24,33^, it is very likely that Portugal’s epidemics played an important role in the early and widespread dissemination of MPXV worldwide. This is supported by our study, as the emergence of some outbreak sublineages, or their early global dissemination, most probably occurred in Portugal. Our study also estimates that only 62% of the true case incidence was observed and that 1.3% of the MSM population was infected during this period, leaving the possibility for similar epidemics to occur. We also consolidated the key role of events or venues with superspreader potential and sexual networks in MPXV transmission and identified the sublineages with the highest impact on outbreak spread in Portugal, while emphasizing their spatiotemporal landscape and international linkage. Our study is an integrative genomic epidemiology analysis in the context of the 2022 worldwide MPXV outbreak. Despite lacking individual network contact data to associate the corresponding genetic subclusters and transmission chains, our combined approach may prove to be crucial to untangle and respond to novel and emerging viral threats, even in contexts as complex as the one faced during the 2022 multi-country mpox outbreak. This study leverages evidence about mpox transmission that can support and guide future public health interventions, including vaccine strategies.

## Methods

This research complies with all relevant ethical regulations. The planning, conduct and reporting of this study was in accordance with the Declaration of Helsinki, as revised in 2013. Ethical approval for the use of surveillance data was not required due to the National Health Authority (Directorate-General of Health) permit to access and use surveillance data for communicable disease outbreak investigations in the public interest. At laboratory level, the Portuguese National Institute of Health (INSA) is the national reference laboratory, being the Portuguese laboratory authorized by the Directorate-General of Health (through the Technical orientation no. 004/2022 of 31 May 2022) to process the samples for identification and genetic characterization of MPXV. All samples subjected to viral genetic characterization were processed in an anonymized fashion. This study was approved by the INSA’s ethical committee ‘Comissão de Ética para Saúde’.

### Epidemiological data collection

Demographic and epidemiological data were collected by the Directorate-General of Health, through SINAVE and the main STD clinics. Data were collected by the attending physician by conducting both face-to-face and phone interviews using the surveillance-standardized case investigation form. Demographic variables that were included were sex and age. Risk practice variables included travel history, contact with a confirmed case, self-identifying as MSM, number of sexual partners, having anonymous and/or multiple sexual partners, engaging in sexual activities with tourists and attending sex venues (sauna, or public or private parties). Countries reported in the travel history inquiry or linked to sexual contacts with tourists, travelers or visitors were referred to as ‘travel-related’ or ‘tourism-related’ countries throughout the manuscript, respectively. Details are presented in Supplementary Table 1.

### Statistical analyses

#### Transmission dynamics

We fitted a discrete-time SEIR model to the reported case data using a daily time step. We assumed an incubation period of 5.6 days (ref. ^44^) and an infectious period of 21 days (ref. ^2,3^). For the number of susceptible cases, we used the estimates for the MSM Portuguese population^45^. The population was assumed to be completely susceptible, and the model was seeded with ten infections at the beginning of the case time series. We estimated a time-varying reproduction number $R(t)$ as a random walk function with a fixed standard deviation of 0.1. The rates of change of each compartment are described in equations (1–4). Here, S, E, I and R represent the susceptible, exposed, infectious and recovered proportions of the population. Additionally, $\alpha$ is the rate of onset of infectiousness, $\sigma$ is the rate of recovery from infection and $\beta \left(t\right)$ is the time-varying transmission rate, calculated as $\sigma R(t)$.

1 $$\frac{{dS}}{{dt}}=-\beta (t)I\left(t\right)S(t)$$

2 $$\frac{{dE}}{{dt}}=\beta I\left(t\right)S\left(t\right)-\,\alpha E(t)$$

3 $$\frac{{dI}}{{dt}}=\,\alpha E\left(t\right)-\,\sigma I(t)$$

4 $$\frac{{dR}}{{dt}}=\,\sigma I(t)$$

The expected number of reported cases at day *t*, $C(t)$ is calculated as shown in equation (5), where $\rho$ is the estimated time-constant reporting rate and $N$ is the size of the population. The model was fit using a negative binomial likelihood with estimated over-dispersion parameter, $\varphi$.

5 $$C\left(t\right)=N\rho \frac{{dI}}{{dt}}$$

The model was fitted in a Bayesian framework using Hamiltonian Monte Carlo No-U-Turn sampling in the CmdStanR package. The model was run for 2,000 iterations with two chains and a warm-up period of 1,000 iterations. Sensitivity results across different infectious periods of 14, 21 and 28 days ($\sigma$) ∈ {0.071, 0.048, 0.036}, with different infection seeds and without accounting for the importations are shown in Supplementary Figs. 1, 2 and 3. We assessed convergence using the R-hat statistic for each parameter.

### DNA Extraction, sequencing and genome consensus generation

All biological samples were received by the Emergency Response and Biopreparedness Unit at INSA and were screened for MPXV using real-time PCR targeting the *rpo18* gene^46^, on a CFX Opus Real-Time PCR System (Biorad), with viral genome sequencing being attempted for available samples with real-time PCR threshold cycle (Ct) ≤ 30. The first ten mpox genome sequences (PT0001 to PT0010) were generated as described in a previous publication^24^. For samples PT0011 to PT0048, with the exception of the DNA extraction (which was conducted using the MagMAX Viral/Pathogen Nucleic Acid Isolation kit in a KingFisher Extractor), the same procedure (Nextera XT library preparation and shotgun metagenomics by 2 × 150 bp paired-end sequencing on an Illumina NextSeq 2000 apparatus) was applied. After the development of an amplicon-based sequencing (PrimalSeq) approach^47^, all subsequent samples (PT0049–PT0595) were processed using an adapted version of this protocol and primer scheme (10.17504/protocols.io.5qpvob1nbl4o/v2). Specifically, DNA amplification was performed using 12.5 µl of NEBNext Q5 Hot Start HiFi PCR Master Mix (New England Biolabs), 3.7 µl of each primer pool (1.5 µM per pool in the final reaction) (in separate reactions), 3.8 µl of nuclease-free water and 5 µl of template DNA in a final reaction volume of 25 µl. PCR amplification conditions were 3 min at 98 °C, followed by 35 cycles of 15 s at 98 °C and 5 min at 63 °C. PCR products of each sample were then pooled and subjected to clean-up with Agencourt AMPure XP (Beckman Coulter, catalog no. A63880) using a 1:1 volume ratio.

Dual-indexed libraries were constructed according to the Nextera XT library preparation guide (Illumina) with minor modifications. Normalization of libraries was performed using a bead-based procedure or a standard procedure according to the concentration values of each library following parallel capillary electrophoresis in the Fragment Analyzer instrument (Agilent). Library pools were denatured and diluted before loading according to the manufacturer’s protocols. Paired-end sequencing (2 × 150 bp) was performed on Illumina NextSeq 550 or NextSeq 2000 instruments. Reference-based genome consensus sequences were obtained using the INSaFLU pipeline v.1.5.2 (https://insaflu.insa.pt/; https://github.com/INSaFLU; https://insaflu.readthedocs.io)^48^, following the same procedure as previously described^24^. Sample details are presented in Supplementary Table 1.

### Phylogenetics and identification of outbreak subclusters

To identify and characterize the subclusters of outbreak-related Portuguese sequences within the framework of the international MPXV genetic diversity, a global IQ-TREE phylogenetic tree was built using the Nextstrain^49^ hMPXV-1 build (https://github.com/nextstrain/monkeypox; commit https://github.com/nextstrain/monkeypox/tree/06ae223e34aff74402e9e4caf5d4322c7c99aad3) over curated genome sequences retrieved from the National Center for Biotechnology Information (NCBI) GenBank Virus collection (https://www.ncbi.nlm.nih.gov/labs/virus/vssi/#). Sequence and metadata curation involved Nextclade, Augur and custom scripts (https://github.com/nextstrain/monkeypox/tree/06ae223e34aff74402e9e4caf5d4322c7c99aad3/ingest). MPXV sublineage classification followed the international nomenclature proposed in https://github.com/mpxv-lineages. As of 2 November 2022, the ‘big’ MPXV Nextstrain public dataset (available for navigation at https://nextstrain.org/groups/neherlab/PT-MPXV-transmission/2022-11-02) included 2,774 sequences, with the B.1 subbranch including 2,721 outbreak-related sequences collected in 28 countries across Europe, Asia, Africa, Oceania, South America and North America (Supplementary Table 1). This 2022 outbreak subtree included 502 Portuguese MPXV genome sequences, of which 494 (representing 18.2% (494 out of 2,714) of the global B.1 sequence dataset, as of 2 November 2022) were used in this study (Supplementary Table 1). Eight Portuguese sequences were excluded from the clustering analysis because of same-patient redundancy and/or uncertainty about patient identification and/or sample collection date). The global outbreak B.1 subtree and respective metadata (Supplementary Dataset 1), including enriched epidemiological data collected for the Portuguese samples (Supplementary Table 1), as described above, were used to identify and characterize all genetic subclusters (defined as any subbranch with at least two sequences diverging from the outbreak basal level by at least one SNP) using ReporTree v.1.0.1 (https://github.com/insapathogenomics/ReporTree)^50^. Specifically, ReporTree was requested to cut the tree at one single threshold level from the outbreak basal level (‘root’) using the ‘root-dist’ method available through the ‘TreeCluster’ analysis mode^51^, and setting a distance unit (‘-d’ argument) corresponding to less than one SNP. ReporTree was also asked to report statistics for all derived subclusters and key metadata variables. A simplified command line string is as follows: *reportree.py -m metadata.tsv -t outbreak_subtree.nwk –analysis treecluster --method-threshold root_dist-1 -d 0.000002 --columns_summary_report metadata_1,metadata_n --metadata2report metadata_1,metadata_n –out reportree*. Advanced and integrative visualization and assessment of the phylogenetic tree, together with genomic, epidemiological and spatiotemporal data was performed with Auspice (https://nextstrain.org/groups/neherlab/PT-MPXV-transmission/2022-11-02). In addition, https://auspice.us/ was used to interactively explore and interpret the identified subclusters (the JSON file, the ‘divergence’ outbreak B.1 tree and metadata with subclusters are available for navigation in Supplementary Dataset 1). In particular, genetic subclusters including at least two Portuguese sequences (‘Portuguese subclusters’) were thoroughly inspected, with the cluster-defining SNP being identified and checked for main homoplasies. The ‘*mutation_profile*’ python script (https://github.com/insapathogenomics/mutation_profile) was applied to screen whether the cluster-defining SNPs follow signatures potentially compatible with APOBEC3-mediated viral genome editing (namely, GA>AA and TC>TT replacements, which were observed in 86% of those SNPs) (Supplementary Table 2). No clustering inferences were taken for the sequences categorized in the ‘outbreak basal level’ (‘root’), because of the outbreak characteristics (see Results) and because one could not exclude the possibility that such ‘under-divergence’ may be due to the lack of genome sequence completeness of some Portuguese and non-Portuguese sequences and/or bioinformatics artefacts (for example, reversions) across the multiple pipelines used worldwide. The identified sublineages were also characterized in terms of timespan; that is, the time (in days) between the earliest and latest detection at both national and international levels. For 21 Portuguese patients, the genotyped sample did not correspond to the patient’s earliest MPXV-positive sample (see Supplementary Table 1). Therefore, to avoid bias in the evaluation of the timespan between the first and latest detection of a given sublineage in Portugal, we used the date of collection of the first PCR-positive sample instead of the date of collection of the first genotyped sample for the timespan analysis (reflected in Fig. 3).

### Reporting summary

Further information on research design is available in the Nature Portfolio Reporting Summary linked to this article.

## Online content

Any methods, additional references, Nature Portfolio reporting summaries, source data, extended data, supplementary information, acknowledgements, peer review information; details of author contributions and competing interests; and statements of data and code availability are available at 10.1038/s41591-023-02542-x.

### Supplementary information

Supplementary Information Supplementary Figs. 1–3

Reporting Summary

Supplementary Tables 1 and 2 Supplementary Table 1. Public MPXV genome sequences (hMPXV-1, Clade IIb, Lineage B.1 and sublineages) used in this study and available metadata for the 494 outbreak PT cases. Supplementary Table 2. Characterization of the Portuguese subclusters according to the size, timespan, international linkage and transmission dynamics.

Supplementary Data 1 Nextstrain JSON file, “divergence” outbreak B.1 tree and metadata with subclusters for navigation in https://auspice.us/.

## Data Availability

MPXV reads mapping to the reference sequence MPXV-UK_P2 (GenBank accession no. MT903344.1) were deposited in the European Nucleotide Archive (ENA) (BioProject accession no. PRJEB53055). Assembled consensus sequences were deposited in the NCBI under the accession numbers detailed in Supplementary Table 1.

## Code availability

Software code used in bioinformatics analysis is available at INSaFLU v.1.5.2 (https://insaflu.insa.pt/; code: https://github.com/INSaFLU/INSaFLU), ReporTree v.1.0.1 (https://github.com/insapathogenomics/ReporTree), get_mutation_profile (https://github.com/insapathogenomics/mutation_profile), Nextstrain (https://github.com/nextstrain/mpox; https://github.com/nextstrain/monkeypox/tree/06ae223e34aff74402e9e4caf5d4322c7c99aad3; https://github.com/nextstrain/monkeypox/tree/06ae223e34aff74402e9e4caf5d4322c7c99aad3/ingest), and MPXV lineages (https://github.com/mpxv-lineages). Software code used in the transmission modelling is available at https://github.com/marianaperezduque/mpox_2022.
