# Supplementary material for: Viral genetic clustering and transmission dynamics of the 2022 mpox outbreak in Portugal
Source: Nat Med. 2023 Sep 11;29(10):2509–17. doi: 10.1038/s41591-023-02542-x (PMC10579057; doi:10.1038/s41591-023-02542-x)
Supplement: Supplementary file 1 — Supplementary Figs. 1–3 [file 41591_2023_2542_MOESM1_ESM.pdf]

nature medicine

Article https://doi.org/10.1038/s41591-023-02542-x

Viral genetic clustering and transmission dynamics of the 2022 mpox outbreak in Portugal

In the format provided by the authors and unedited

Supplementary Figures

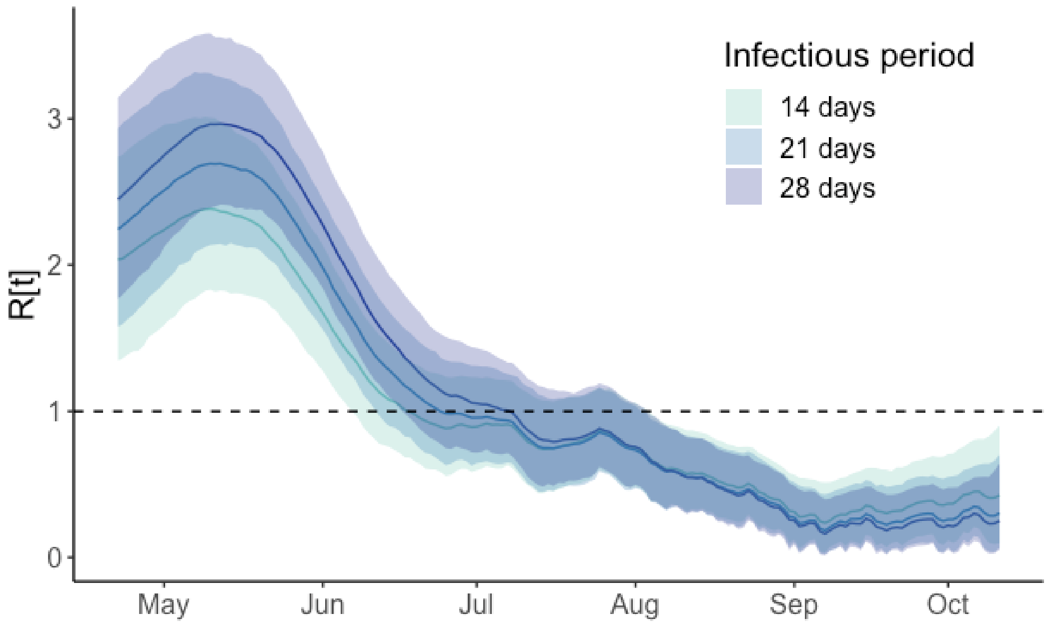

Supplementary Figure 1. Sensitivity analysis of the infectious period on model parameters estimates of MPVX outbreak in Portugal, April-October 2022. Estimated time-varying reproduction number *R(t)* using Hamiltonian Monte Carlo No-U-Turn sampling across 14, 21 (our model's prior) and 28 days of infectiousness[2]. The solid red (14 days infectiousness), green (21 days infectiousness), and blue (28 days infectiousness), lines show the median R(t) estimates for each time point. The red (14 days infectiousness), green (21 days infectiousness), and blue (28 days infectiousness) bands show the corresponding 95% credible interval (CrI) of the median R(t) estimates. The horizontal black dashed line at value 1 shows the threshold for continued transmission. An infectious period of 28 days led to a maximum estimated *R(t)* of 2.96 (95%CrI: 2.41-3.56) on May 12th. Using an infectious period of 14 days we estimated a maximum *R(t)* on May 10th of 2.38 (95%CrI: 1.83-3.01).

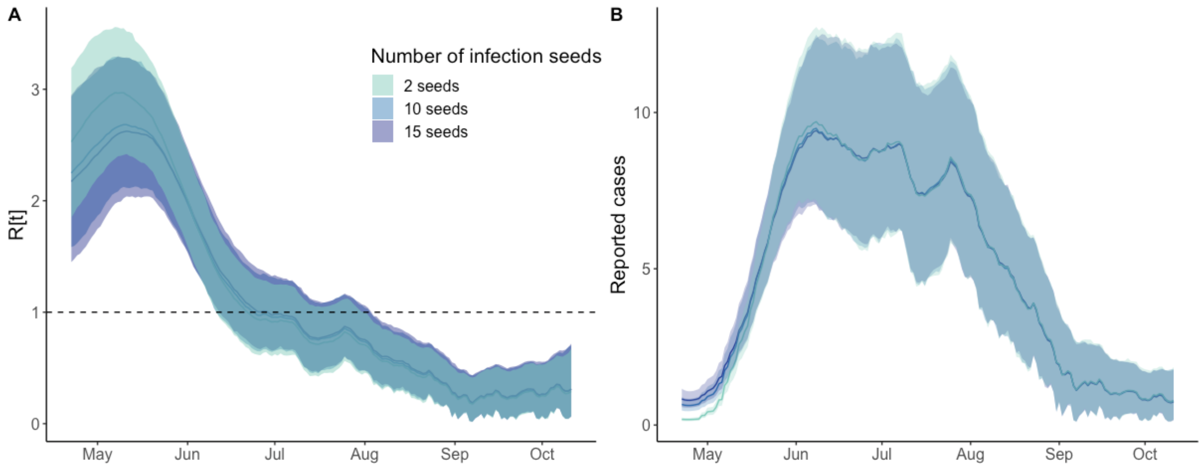

Supplementary Figure 2. Sensitivity analysis of the number of infection seeds on model parameters estimates and model fit of MPVX outbreak in Portugal, April-October 2022. A. Estimated time-varying reproduction number R(t) using Hamiltonian Monte Carlo No-U-Turn sampling across 2, 10 (our model's prior) and 15 infection seeds. The solid blue (2 infectious seeds), red (10 infectious seeds), and green (15 infectious seeds) lines show the median R(t) estimates for each time point. The blue (2 infectious seeds), red (10 infectious seeds) and green bands (15 infectious seeds), show the corresponding 95% credible interval (CrI) of median R(t) estimates. The horizontal black dashed line at value 1 shows the threshold for continued transmission. A seed of 2 infections led to a maximum estimated *R(t)* of 2.97 (2.38-3.56) on May 7th. Using 15 infection seeds we estimated a maximum *R(t)* on May 11th of 2.62 (95%CrI: 2.04-3.29). B shows the model fit (line) for the estimated median number of reported cases and the corresponding 95% CrI (band) with 2 (blue), 10 (red) and 15 (green) infections seeds. The estimated reporting rate for 2 infection seeds is 0.77 (95% CrI 0.61-0.91) with 1101 estimated infections (95%CrI: 685-1671). The estimated reporting rate for 15 infection seeds is 0.56 (95% CrI 0.37-0.77) with 1532 estimated infections (95%CrI: 893-2584).

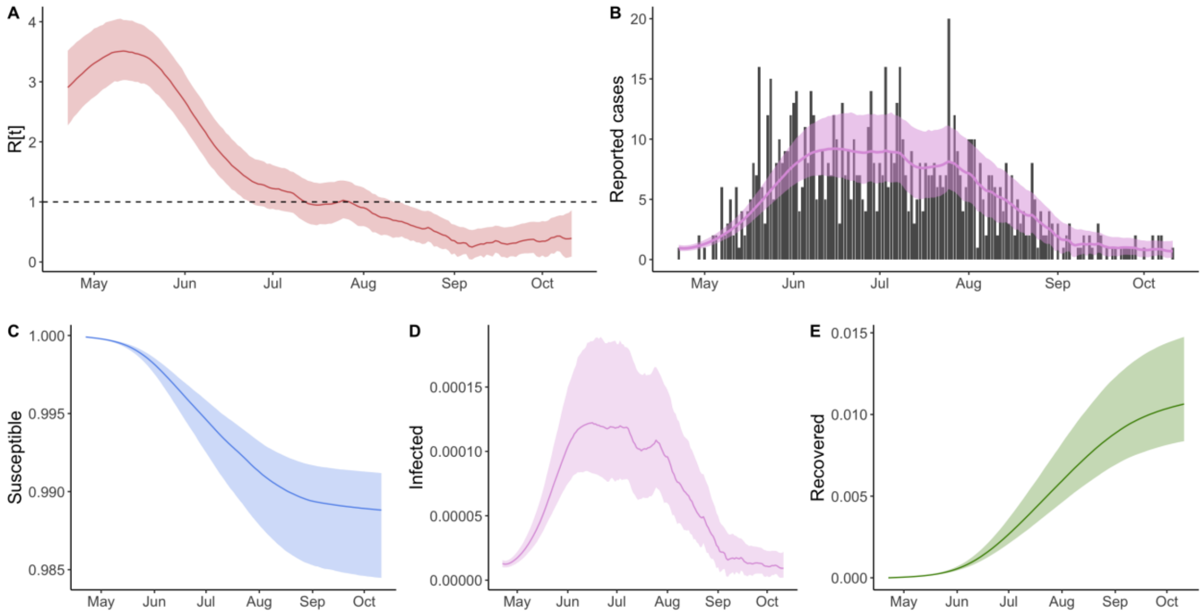

Supplementary Figure 3. Sensitivity analysis without travel history on model parameters estimates and model fit of MPVX outbreak in Portugal, April-October 2022. A shows the estimated time-varying reproduction number R(t) using Hamiltonian Monte Carlo No-U-Turn sampling. The solid red line shows the median R(t) estimates for each time point. The red band shows the corresponding 95% credible interval (CrI) of the median R(t) estimates. The horizontal black dashed line at value 1 shows the threshold for continued transmission. **B** shows the model fit against the epidemic curve of MPXV cases. The solid pink line shows the estimated median of case reported for each time point. The pink band shows the corresponding 95%CrI of the model fit for the estimated median of cases reported. The estimated reporting rate was 0.71 (95%CrI: 0.52-0.87) with 1188 estimated infections (95%CrI: 773-1825). **C, D and E** lines and bands (blue, pink and green) show the estimated proportion and the corresponding 95% CrI of the MSM population to be susceptible, infected and recovered, respectively, using a serial interval of 5.6 days and with 10 infection seeds. The model estimates the epidemic peaked on 16th of June with 13 infections (95%CrI: 9-20). The estimated MSM population infected was 1.1% (95%CrI: 0.72-1.70). MPXV case notification data (n=865) were obtained from the Portuguese National Epidemiological Surveillance System.
