# Supplementary material for: Viral genetic clustering and transmission dynamics of the 2022 mpox outbreak in Portugal
Source: Nat Med. 2023 Sep 11;29(10):2509–17. doi: 10.1038/s41591-023-02542-x (PMC10579057; doi:10.1038/s41591-023-02542-x)
Supplement: Supplementary file 2 — Reporting Summary [file 41591_2023_2542_MOESM2_ESM.pdf]

nature portfolio

Corresponding author(s): João Paulo Gomes

Last updated by author(s): Aug 3, 2023

Reporting Summary

Nature Portfolio wishes to improve the reproducibility of the work that we publish. This form provides structure for consistency and transparency in reporting. For further information on Nature Portfolio policies, see our Editorial Policies and the Editorial Policy Checklist.

Statistics

For all statistical analyses, confirm that the following items are present in the figure legend, table legend, main text, or Methods section.

n/a	Confirmed	
☐	☒	The exact sample size (*n*) for each experimental group/condition, given as a discrete number and unit of measurement
☒	☐	A statement on whether measurements were taken from distinct samples or whether the same sample was measured repeatedly
☒	☐	The statistical test(s) used AND whether they are one- or two-sided<br>*Only common tests should be described solely by name; describe more complex techniques in the Methods section.*
☒	☐	A description of all covariates tested
☒	☐	A description of any assumptions or corrections, such as tests of normality and adjustment for multiple comparisons
☐	☒	A full description of the statistical parameters including central tendency (e.g. means) or other basic estimates (e.g. regression coefficient) AND variation (e.g. standard deviation) or associated estimates of uncertainty (e.g. confidence intervals)
☒	☐	For null hypothesis testing, the test statistic (e.g. *F*, *t*, *r*) with confidence intervals, effect sizes, degrees of freedom and *P* value noted<br>*Give P values as exact values whenever suitable.*
☒	☐	For Bayesian analysis, information on the choice of priors and Markov chain Monte Carlo settings
☒	☐	For hierarchical and complex designs, identification of the appropriate level for tests and full reporting of outcomes
☒	☐	Estimates of effect sizes (e.g. Cohen's *d*, Pearson's *r*), indicating how they were calculated

Our web collection on statistics for biologists contains articles on many of the points above.

Software and code

Policy information about availability of computer code

Data collection	No software was used.
Data analysis	Codes: INSaFLU v.1.5.2 (https://insaflu.insa.pt/; code: https://github.com/INSaFLU/INSaFLU); ReporTree v.1.0.1 (https://github.com/insapathogenomics/ReporTree); get_mutation_profile (https://github.com/insapathogenomics/mutation_profile); Nextstrain (https://github.com/nextstrain/mpox ; https://github.com/nextstrain/monkeypox/tree/06ae223e34aff74402e9e4caf5d4322c7c99aad3; https://github.com/nextstrain/monkeypox/tree/06ae223e34aff74402e9e4caf5d4322c7c99aad3/ingest); Mpox lineages (https://github.com/mpxv-lineages); Transmission modelling (https://github.com/marianaperezduque/mpox_2022)

For manuscripts utilizing custom algorithms or software that are central to the research but not yet described in published literature, software must be made available to editors and reviewers. We strongly encourage code deposition in a community repository (e.g. GitHub). See the Nature Portfolio guidelines for submitting code & software for further information.

Data

Policy information about availability of data

All manuscripts must include a data availability statement. This statement should provide the following information, where applicable:

- Accession codes, unique identifiers, or web links for publicly available datasets
- A description of any restrictions on data availability
- For clinical datasets or third party data, please ensure that the statement adheres to our policy

Mpox reads mapping to the reference sequence MPXV-UK_P2 (GenBank accession #MT903344.1) were deposited in the European Nucleotide Archive (ENA) under the BioProject accession no. PRJEB53055 (http://www.ebi.ac.uk/ena/data/view/PRJEB53055). Assembled consensus sequences were deposited in the National Center for Biotechnology Information (NCBI) under the accession numbers detailed in Supplementary Table 1.

Human research participants

Policy information about studies involving human research participants and Sex and Gender in Research.

Reporting on sex and gender	This study is not based on the recruitment of human participants.
Population characteristics	This study is not based on the recruitment of human participants. It focus the use of viral genomic data (obtained through the analysis of samples derived from the diagnostic of mpox disease) combined with epidemiological data to monitor the spread of a disease throughout an entire country.
Recruitment	Not applicable, as this is a retropective analysis of infected cases on behalf of a massive outbreak (i.e., recruitment could not be part of the study design).
Ethics oversight	Ethical approval for the use of surveillance data was not required due to the National Health Authority (Directorate-General of Health) permit to access and use surveillance data for communicable disease outbreak investigations in the public interest. At laboratory level, the Portuguese NIH (INSA) is the national reference laboratory, being the Portuguese laboratory authorizsed by the Directorate-General of Health (through the Technical orientation nº 004/2022 of May 31st 2022) to process the samples for identification and genetic characterization of MPXV. All samples subjected to viral genetic characterization were processed in an anonymized fashion. This study was approved by the INSA's ethical committee "Comissão de Ética para Saúde".

Note that full information on the approval of the study protocol must also be provided in the manuscript.

Field-specific reporting

Please select the one below that is the best fit for your research. If you are not sure, read the appropriate sections before making your selection.

☒ Life sciences ☐ Behavioural & social sciences ☐ Ecological, evolutionary & environmental sciences

For a reference copy of the document with all sections, see nature.com/documents/nr-reporting-summary-flat.pdf

Life sciences study design

All studies must disclose on these points even when the disclosure is negative.

Sample size	We performed viral whole genome sequencing (WGS) from all samples for which we detected a viral load suitable to follow to WGS (defined by the real-time PCR threshold cycle values).
Data exclusions	Samples presenting a very low viral load (defined by the real-time PCR threshold cycle values) were excluded.
Replication	Not applicable in this sense, considering the study nature. Nevertheless, specific experimental reproducibility is ensured both by the results' parallelism obtained with the different samples and by the high depth of coverage in sequencing data.
Randomization	Not applicable, as this is a retropective analysis of infected cases on behalf of a massive outbreak (i.e., randomization could not be part of the study design).
Blinding	Blinding is not applicable. All epidata that complements the viral genomic data is anonymized and all samples were analysed strictly for genomic purposes (i.e., all samples with viral load compatible with sequencing).

Reporting for specific materials, systems and methods

We require information from authors about some types of materials, experimental systems and methods used in many studies. Here, indicate whether each material, system or method listed is relevant to your study. If you are not sure if a list item applies to your research, read the appropriate section before selecting a response.

Materials & experimental systems

n/a	Involved in the study
☒	☐ Antibodies
☒	☐ Eukaryotic cell lines
☒	☐ Palaeontology and archaeology
☒	☐ Animals and other organisms
☒	☐ Clinical data
☒	☐ Dual use research of concern

Methods

n/a	Involved in the study
☒	☐ ChIP-seq
☒	☐ Flow cytometry
☒	☐ MRI-based neuroimaging
